# Increased patient satisfaction by integration of palliative care into geriatrics—A prospective cohort study

**DOI:** 10.1371/journal.pone.0287550

**Published:** 2023-06-22

**Authors:** Maria E. C. Schelin, Carl Johan Fürst, Birgit H. Rasmussen, Christel Hedman

**Affiliations:** 1 Institute for Palliative Care, Lund University and Region Skåne, Lund, Sweden; 2 Department of Clinical Sciences Lund, Lund University, Lund, Sweden; 3 Faculty of Medicine, Department of Health Sciences, Lund University, Lund, Sweden; 4 Department of Molecular Medicine and Surgery, Karolinska Institutet, Stockholm, Sweden; 5 R & D department, Stockholms Sjukhem Foundation, Stockholm, Sweden; Scuola Superiore Sant Anna PISA, ITALY

## Abstract

**Background:**

Integration of oncology and palliative care has been shown to increase quality of life in advanced disease. To meet the needs of the growing older population, integration of palliative care and geriatrics has been proposed but scarcely described.

**Objectives:**

The aim of this study was to integrate palliative care into geriatrics by a structured care guide, the Swedish Palliative Care Guide, and to evaluate its effect on patient satisfaction, health-related quality of life and symptom burden, compared to a control group.

**Methods:**

Geriatric in-patients over 65 years of age were included in the study, those with cognitive impairment were excluded. Data was collected before (baseline) and after the implementation (intervention) of the Swedish Palliative Care Guide. Patient satisfaction was evaluated two weeks after discharge with questions from a national patient survey. Health-related quality of life was measured with EQ-5D-3L and symptom burden with Edmonton Symptom Assessment Scale.

**Results:**

In total, 400 patients were included, 200 in the baseline- and intervention group, respectively. Mean age was 83 years in both groups. Patient satisfaction was significantly higher in nine out of ten questions (p = 0.02-<0.001) in the intervention group compared to baseline. No differences between the groups were seen in health-related quality of life or symptom burden.

**Conclusion:**

A significant effect on patient satisfaction was seen after implementation of the Swedish Palliative Care Guide in geriatric care. Thus, integration of palliative care and geriatrics could be of substantial benefit in the growing population of older adults with multimorbidity and frailty.

## Introduction

The population in Europe and worldwide is getting older [1] and the lives of the aging people are affected by multimorbidity, functional dependency, cognitive decline and increasing frailty [2,3]. The needs of geriatric patients differ from the needs in younger patients regarding prevalence and intensity of symptoms, needs of hospitalisation, decision-making, and support to families and are too often unmet [4]. Thus, the healthcare system needs tailored, holistic, patient-centred care to meet the demands of the aging population.

To achieve this, integration of palliative care and geriatrics, i.e. geriatric palliative care, has been proposed [5,6]. While geriatrics is a medical speciality focusing on the healthcare of older people, the aim of palliative care is to treat physical and psychological symptoms and to address existential issues to improve quality of life independently of diagnosis or age [7]. Moreover, it increases patient-centred care and communication and could improve decision-making in end-of-life care [7,8]. Palliative care is known to decrease health-care utilisation [9] including hospital referral [10], and enable more people to die at home [11], which is a well-known priority also among older people [12,13].

Integration of palliative care into oncology has been shown to decrease symptom burden, increase quality of life and even prolong life [14,15]. More diverse results are seen in non-cancer diseases, like COPD [16], heart failure [17] and neurological diseases [18], with modest effect on symptom burden and no significant increase in quality of life [19]. One study on integration of palliative care into geriatrics showed that implementation of a palliative care programme in end-of-life care improved comfort around dying assessed by nurses but decreased satisfaction with care reported by family members [20]. To date there is no well-described methods of how to integrate palliative care into geriatrics [21].

The Swedish Palliative Care Guide (S-PCG) was aimed at facilitating integration of a palliative care perspective into the healthcare system both during end-of-life care but also earlier in the disease trajectory. It was developed according to the Medical Research Council framework and national and international palliative care guidelines [22]. S-PCG provides an interprofessional guidance for evidence-based and patient-centred palliative care, independent of diagnosis, healthcare setting and remaining lifetime. It consists of four parts; Part 1 for identification of palliative care needs, Part 2 to support provision of palliative care regardless of time left in life, Part 2^D^ is for support during the last days of life and lastly, Part 3 is a guide for the care after death and bereavement support [23]. S-PCG provides guidance and facilitates documentation regarding assessment of symptoms and function of daily life, communication and decision-making, patients´ preferences including wishes and priorities, social context, planning of care, the care of the dying person and care of the deceased and bereavement support [22,24]. Although used in more than 400 units all over Sweden, mainly in elderly care, there are no studies of the effect of implementing the S-PCG.

### Aim

The aim of this study was to evaluate the effect of using the structured care guide S-PCG Part 2 on patients´ satisfaction, health-related quality of life (HRQOL) and symptom burden and also to assess satisfaction of care from the next of kins’ perspective.

## Materials and methods

### Study design and study population

This single-centre prospective cohort study with a before-and-after design was performed between April 2019 and December 2020 in a in a hospital with one of the largest geriatric clinics in Sweden. In this study we implemented and studied the effect of Part 2 of the S-PCG [23]. The study was divided into three parts; 1) first as a baseline (pre-intervention/control) when patients were consecutively included April to September 2019, 2) teaching of staff and clinical implementation of S-PCG, September to December 2019 and 3) the intervention, when patients were consecutively included as S-PCG was in clinical use, January to December 2020.

Inclusion criteria were age ≥65 years, hospital stay ≥5 days, discharge to home, including nursing home but excluding hospital. The same patient was allowed to be included both in the pre-intervention and the intervention part of the study if they indeed were admitted in both periods. Exclusion criteria were cognitive impairment (assessed as a dementia diagnosis, research–or clinical staff recognizing cognitive impairment, or delirium from medical records or a clinical judgement) or a COVID-19 diagnosis. Patients with cognitive impairment were excluded, as the method was to collect data through questionnaires sent to patients two weeks after discharge, which is not suitable for patients with cognitive impairment. All patients meeting the inclusion criteria were asked for participation consecutively by the same researcher (CH). During the pre-intervention period and in the beginning of the intervention period all patients were asked for inclusion in person on the geriatric ward. Due to the COVID-19 pandemic, from March to December 2020, patients were recruited by a phone call after discharge. All included patients were given verbal and written information and signed an informed consent statement. Two to three weeks after discharge patients were sent questionnaires about their satisfaction with care, quality of discharge, symptom burden and HRQOL. Non-respondents were contacted by phone and up to two reminders were sent to them.

Patients were asked about permission for the researchers to contact a next of kin. Next of kin were then approached by mail and asked about their satisfaction of the patient´s care. Non-respondents received two reminders. After the COVID-19 pandemic started (March 2020) no next of kin were approached, as they could not evaluate the quality of care due to visiting restrictions. The study was approved by the Ethical Review Board in Sweden (DNR 2019–01689, 26^th^ March 2019).

### The education and implementation process

The implementation process of the S-PCG used the methodology outlined during the development of the guide at Lund University [24]. The implementation started with a whole day introductory education about the background and aim of S-PCG and how to use it in practice. This education included information and discussion about S-PCG, a basic introduction to palliative care and why a palliative care approach is important in geriatric patients. It also included education in symptom assessment, communication with the patients´ about wishes and priorities, the understanding of their disease and focus and content of care, as these are important parts of the S-PCG. All professions were included in the education and implementation of S-PCG. To ensure structured documentation an adoption of the documentation procedure took place before starting the implementation of S-PCG. The education was followed by a four-month period of implementation when the S-PCG was used among patients, in the beginning in some patients and in the end of the period in all patients excluding only those with cognitive impairment. Nurses used the S-PCG when they met the patients and assessed symptoms, asked about wishes and priorities, their understanding of their disease and their relationships to family and other important persons. Function of daily life was assessed by physiotherapists and occupational therapists. Medical decisions, like changes in medication, possible treatment limitations and discussion about focus and content of care were made by physicians. The result of these interactions was documented in the S-PCG document or the medical records (depending on clinical documentation routine) with the aim of a more person-centred care focusing on each patient’s individual needs. In addition, short palliative care educations of about 30 minutes were held every four to six weeks covering topics raised by the health-care professionals (HCPs).

### The intervention period

During the intervention period patients were included twice a week at the geriatric ward. After the COVID-19 outbreak the inclusion of patients was made by phone after discharge but the visits on the ward by the principal investigator continued approximately once a week primarily to answer HCPs´ questions. The above-mentioned palliative care educations were also continued and during the educations HCPs could discuss experiences regarding the use of the S-PCG.

### Data collection and categorization

#### Sociodemographic variables, comorbidities and frailty

In the questionnaire patients were asked to report co-living and level of education. The main diagnosis for the current stay and length of stay were retrieved from medical records. Frailty was assessed by the Hospital Frailty Risk Score based on the diagnoses (ICD-10 codes) in the medical records during the hospital stay [25]. Risk assessment of the patients was determined by the Norton Score [26], the Mini Nutritional Assessment Short-Form (MNA-SF) [27] and the Downton Fall Risk Index [28] at admission. Next of kin were asked about gender and their relation to the patient.

#### Satisfaction with care and discharge quality

To evaluate satisfaction with care we used selected questions from the National patient survey [29] in Sweden. The National patient survey was launched in 2009 and the core questions were based on the Picker survey [30]. The survey contains 38 questions about the hospital stay. For this study we selected 10 questions, focusing on patient satisfaction and patient-centered care, thus including questions about decision-making, participation in care, communication, and information. The questions were scored on a 5-point Likert-scale: “Never”, “Seldom”, “Sometimes”, “Often” and “All of the time”. To evaluate satisfaction with care experienced by next of kin we used the same 10 questions as for patients but reformulated them linguistically to suit next of kin. Discharge quality was measured with the “Discharge Care Experiences Survey Modified” (DICARES-M) [31], a questionnaire about discharge quality in elderly patients developed in Norway. It contains 11 items scored on a 5-point Likert-scale ranging from 1 =“not at all to” 5 =“to a very large extent” and a total sample mean score.

#### Health-related quality of life and symptom burden

HRQOL was assessed with the Swedish version of EQ-5D-3L measuring impairment in five aspects of health: mobility, self-care, usual activities, pain/discomfort and anxiety/depression on a three-grade scale from 1 =“no problems” to 3 =“extreme problems” [32], this tool is validated among the elderly [33]. The EQ-5D VAS score was obtained by asking the patients to rate their health on a vertical scale ranging from 0 to 100, where 0 = “worst imaginable health” and 100 = “best imaginable health”.

Symptom burden was assessed with Edmonton Symptom Assessment Scale (ESAS) [34], measuring intensity of nine common symptoms: pain, tiredness, drowsiness, nausea, lack of appetite, shortness of breath, depression, anxiety and feeling of well-being. The scale ranges from 0 =“no symptom” to 10 =“worst possible symptom severity”.

### Statistical analysis

Patient characteristics were described by standard descriptive statistics. Statistical differences in questionnaires were tested with a two-way t-test. EQ-5D-3L data was analysed with the Swedish value set for EQ-5D health states [35]. As statistical significance alone is not sufficient when evaluating HRQOL data, minimal clinically important difference (MCID) was used for EQ-5D-3L. For EQ-5D index a difference >0.06 [36] and for EQ-5D VAS a difference >7 is interpreted as MCID [37]. When including 200 patients we have 80% power to find a difference of 0.2 points (scale 0–5) if our cohort has a standard deviation of 0.7 and alpha = 0.05. Regarding missing data, we used complete case analysis since the level of missing data was very low. Missing data are presented as footnotes. The statistical software Stata v17.0 (Stata Corp., College Station, TX) was used for all analyses.

## Results

### Characteristics of patients and next of kin

**Fig 1:**
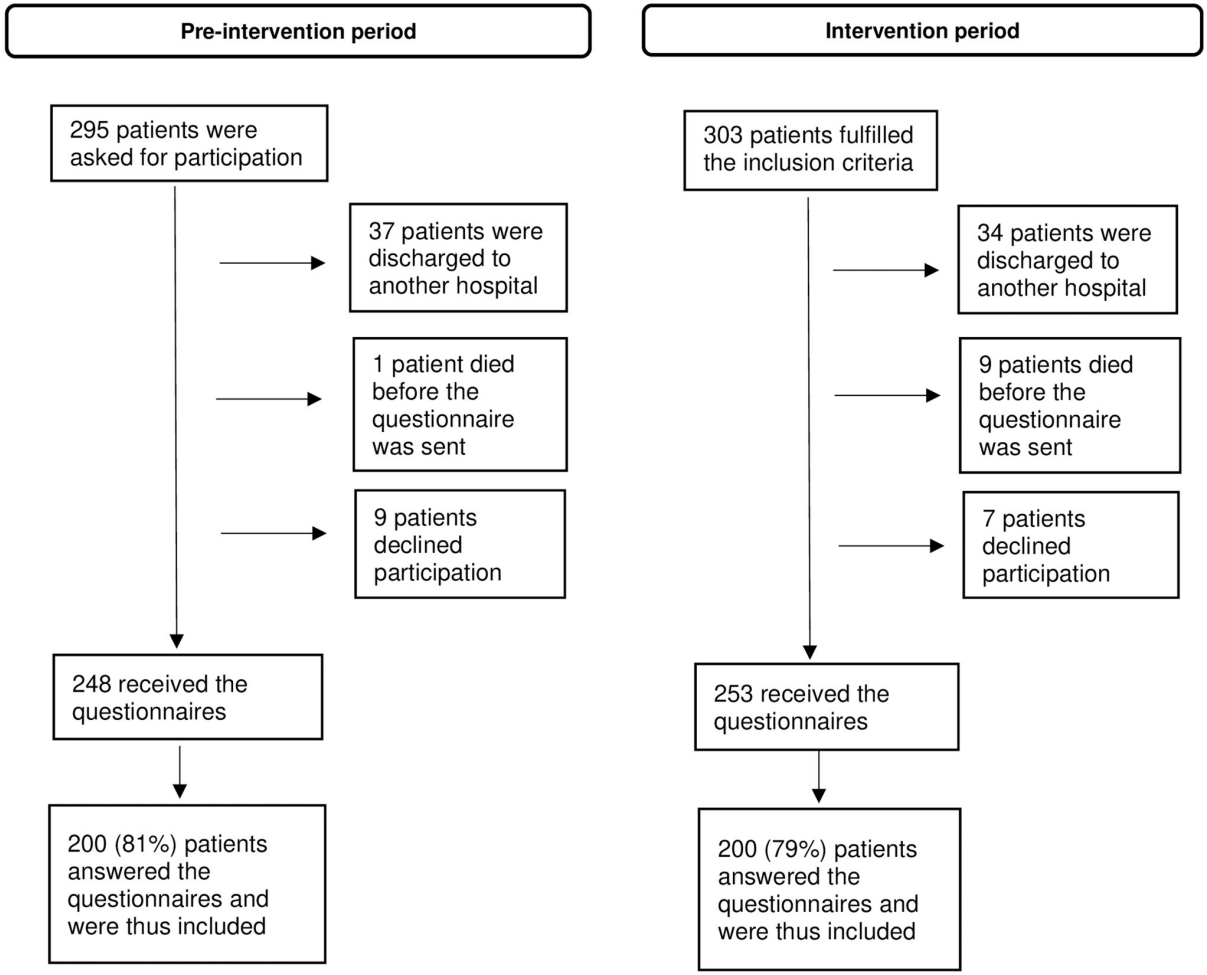
Flowchart for inclusion of patients in the study.

Of the 248 eligible patients in the pre-intervention group 200 answered the questionnaires (81%) and in the intervention group corresponding numbers were 253 and 200 (79%) patients (Fig 1). The majority in pre-intervention and intervention groups were women (65% and 63%, p = 0.76) and mean age was 82.5 and 83 years respectively (p = 0.53). Further, no statistical differences were found in educational level, or marital status between the groups. The most common reasons for geriatric care were trauma/musculoskeletal diseases (34% in both groups) and infections (22% in pre-intervention and 21% in intervention group), see Table 1. Length of hospital stay decreased from 9.8 to 8.8 days between the pre-intervention and intervention period, which is in line with the decrease of hospital stay in the geriatric clinic in general during the study period 2019–2020. No differences between the groups were found inHospital Frailty Risk Score, the Norton Score, the MNA-SF or the Downton Fall Risk Index (Table 1). One patient was included both in the pre-intervention and the intervention group.

**Table 1:** A comparison of patient and next of kin characteristics between pre-intervention group and intervention group.

**Patient characteristics**		**Pre-intervention (n = 200)** n (%)	**Intervention (n = 200)** n (%)	p-value
**Age** mean (range)				
		82.5 (65–101)	83.0 (65–102)	0.53
**Gender**				
	Female	129 (65)	126 (63)	0.76
**Educational level**				
	Low (Elementary school < 9 years)	47 (24)	44 (23)	0.40
	Medium (Upper secondary School 10–12 years)	57 (29)	45 (23)	
	High (University >12 years)	93 (47)	103 (54)	
**Marital status**				
	Married/co-living	66 (37)	73 (36.5)	0.59
	Other	133 (67)	127 (63.5)	
**Main diagnoses**				
	Cancer	5 (3)	10 (5)	
	Infection	44 (22)	41 (21)	
	Respiratory disease	9 (5)	11 (6)	
	Cardio- and cerebrovascular disease	33 (17)	38 (19)	
	Trauma and musculoskeletal disease	67 (34)	67 (34)	
	Gastrointestinal disease	20 (10)	16 (8)	
	Other	22 (11)	17 (9)	
**Hospital Frailty Risk Score** (Mean (CI)		2.16 (1.86–2.45)	2.16 (1.87–2.44)	0.50
**Norton score** (Mean (CI)		16.30 (16.04–16.56)	16.72 (16.37–17.07)	0.07
**MNA-SF** (Mean (CI)		9.88 (9.55–10.21)	10.07 (9.71–10.42)	0.77
**Downton Fall Risk Index** (Mean (CI)		4.07 (3.85–4.29)	4.02 (3.76–4.28)	0.38
**Length of hospital stay in days** mean, range		9.8 (5–23)	8.8 (5–29)	0.004
Discharge destination				
	Home	190 (98)	194 (98)	0.40
	Nursing home	4 (2)	3 (2)	
**Characteristics of next-of-kin**		**Pre-intervention** (n = 86) n (%)	**Intervention** (n = 42) n (%)	p-value
**Age** mean (range)				
		65.7 (40–89)	66.1 (40–87)	0.83
**Gender**				
	Female	54 (63)	30 (71)	0.33
**Relation to patient**				
	Spouse	35 (41)	18 (43)	0.96
	Child	37 (43)	17 (41)	
	Other	14 (16)	7 (17)	

MNA-SF = Mini Nutritional Assessment Short-Form.

Missing data: Educational level: N = 3 pre-intervention N = 8 intervention, marital status: N = 1 pre-intervention, discharge destination N = 6 pre-intervention N = 3 intervention.

There was no difference in age between responders and non-responders in either group (82.5 vs 84.0 years, p = 0.23, for pre-intervention and 83.0 vs 83.2 years, p = 0.83, for intervention). Among the responders there were less women in the pre-intervention group compared to the responders (women 64.5% vs 81.3%, p = 0.03) with no difference in gender in the intervention group (women 63.0% vs 73.6%, p = 0.15).

Because of the visiting restrictions during the COVID-19 pandemic only 42 next of kin were included in the intervention group, compared to 86 in pre-intervention. The majority were children or spouse to the patient (Table 1).

### Satisfaction with care and discharge quality

In nine out of ten questions measuring satisfaction with care the results significantly improved in the intervention group (p<0.001–0.023). The only question not showing significant improvement was if the patient felt safe on the ward (p = 0.173). This question had a mean of 4.45 in pre-intervention compared to 4.67 out of 5 in the intervention group (p = 0.173) (Table 2). Regarding next of kins´ experiences about satisfaction with care one of the questions showed significant improvement during the intervention period (p<0.01) and there was also a numerical increase in all other questions in the intervention group compared to the pre-intervention group, which however didn´t reach statistical significance (S1 Table).

**Table 2:** Patient satisfaction with care, measured with ten questions from the Swedish National patient survey. Results were compared between pre-intervention and intervention period.

Patients	Pre-intervention (n = 200) Mean (CI)	Intervention (n = 200) Mean (CI)	p-value
Did the HCPs consider your experience of your own state of health?^1^	3.53 (3.37–3.70)	3.86 (3.71–4.01)	**<0.01**
If you, during your stay, spoke to several HCPs, were they consistent regarding their communication?^2^	3.83 (3.67–3.99)	4.07 (3.94–4.21)	**0.02**
Did you have the possibility to ask the questions you wanted?^3^	3.85 (3.69–4.01)	4.16 (4.02–4.30)	**<0.01**
If you asked HCPs questions, did you receive answers you understood?^4^	4.10 (3.96–4.24)	4.33 (4.20–4.46)	**0.02**
Did HCPs involve you in decisions regarding your care/treatment?^5^	3.33 (3.14–3.52)	3.75 (3.56–3.94)	**<0.01**
Did you receive enough information about your care/treatment?^6^	3.50 (3.31–3.69)	3.84 (3.67–4.01)	**<0.01**
Did you receive enough information where to turn if you needed help or had additional questions after your stay?^7^	3.20 (2.98–3.41)	3.58 (3.36–3.79)	**0.02**
Do you think that the HCPs on the ward coordinated your contacts with the health care in the extent you needed? ^8^	3.42 (3.21–3.63)	4.04 (3.88–4.21)	**<0.01**
Did the personal take into consideration your home/family situation when planning your discharge?^9^	3.68 (3.46–3.90)	4.02 (3.83–4.21)	**0.02**
Did you feel safe during your stay in the ward?^10^	4.45 (4.43–4.69)	4.67 (4.58–4.77)	0.17

CI: Confidence interval.

HCP: Health-care professional.

Missing data:

^1^N = 13,

^2^N = 16,

^3^N = 8,

^4^N = 5,

^5^N = 11

^6^ N = 5,

^7^N = 9,

^8^N = 13,

^9^N = 12,

^10^N = 3.

Discharge quality was measured with the eleven item DICARES-M questionnaire. One item, about patients notifying HCPs about important issues, showed significant improvement (p = 0.025) in the intervention group. In the remaining ten items no significant changes were found. Further, no difference was found in the total score between the groups (Table 3).

**Table 3:** Discharge quality among patients, measured with the Discharge Care Experiences Survey Modified (DICARES-M) questionnaire. Results were compared between pre-intervention and intervention period.

	Pre-intervention (n = 200) Mean (CI)	Intervention (n = 200) Mean (CI)	p-value
I have felt stressed*^,^^1^	3.87 (3.70–4.04)	3.90 (3.75–4.06)	0.75
I have felt blue*^,^^2^	3.73 (3.57–3.90)	3.77 (3.61–3.93)	0.75
I have experienced problems in performing daily activities (e.g. personal hygiene, getting dressed, cooking)*^,^^3^	3.48 (3.30–3.66)	3.67 (3.49–3.85)	0.15
I have experienced problems in getting sufficient nutrition*^,^^4^	3.93 (3.77–4.10)	4.09 (3.92–4.25)	0.21
In connection with being discharged, I had an opportunity to notify hospital personnel about what I thought was important^5^	2.60 (2.42–2.78)	2.89 (2.71–3.08)	**0.03**
When I was discharged from the hospital, I understood thoroughly the purpose of taking my medication^6^	4.07 (3.91–4.24)	3.98 (3.81–4.16)	0.48
I got information about effects and side effects of my medications^7^	2.21 (2.01–2.39)	2.37 (2.18–2.55)	0.24
When I was discharged from the hospital. I had a good understanding of my responsibility in terms of looking after my health^8^	3.88 (3.71–4.04)	3.85 (3.69–4.01)	0.84
I have experienced problems in understanding the instructions I received when I was discharged from hospital*^,^^9^	4.40 (4.26–4.55)	4.55 (4.42–4.68)	0.14
I have experienced problems in following the instructions I received when discharged from the hospital*^,^^10^	4.43 (4.28–4.56)	4.45 (4.31–4.59)	0.899
I felt I was discharged too early*^,^^11^	4.08 (3.89–4.26)	4.11 (3.93–4.29)	0.79
**Total sample mean score**	3,94 (3.83–4.06)	4.05 (3.94–4.16)	0.21

*Negative statements were inverted to a positive scale.

CI: Confidence interval.

Missing:

^1^N = 4,

^2^N = 7,

^3^N = 7,

^4^N = 5,

^5^N = 9,

^6^N = 11,

^7^N = 13,

^8^N = 18,

^9^N = 23,

^10^N = 23,

^11^N = 5.

### Health-related quality of life and symptom burden

No significant differences were found between pre-intervention and intervention group in the EQ-5D index. The EQ-5D VAS was statistically significantly improved from 51 to 55 (p = 0.049), but the improvement was not clinically significant [36]. Further, no significant differences were found between pre-intervention and intervention group regarding symptoms (Table 4).

**Table 4:** Health-related quality of life, measured with EQ-5D-3L and symptom burden, measured with Edmonton Symptom Assessment Scale (ESAS).

	Pre-intervention (n = 200) Mean, (CI)	Intervention (n = 200) Mean, (CI)	p-value
**Health-related quality of life measured with EQ-5D-3L**			
EQ-5D Index	0.72 (0.70–0.74)	0.73 (0.71–0.75)	0.580
EQ-5D VAS	51.03 (48.16–53.90)	55.19 (52.22–58.16)	**0.048**
**Symtomburden measured with ESAS**			
Pain^1^	3.74 (3.33–4.16)	3.72 (3.30–4.15)	0.95
Tiredness^2^	5.20 (4.83–5.57)	5.46 (5.10–5.83)	0.32
Drowsiness^3^	4.18 (3.78–4.58)	4.22 (3.81–4.63)	0.89
Nausea^4^	1.17 (0.87–1.48)	0.96 (0.71–1.21)	0.28
Lack of appetite^5^	2.89 (2.49–3.30)	2.61 (2.20–3.02)	0.33
Shortness of breath^6^	3.33 (2.91–3.76)	3.57 (3.11–4.03)	0.45
Depression^7^	3.30 (2.88–3.71)	3.05 (2.64–3.46)	0.41
Anxiety^8^	2.27 (1.88–2.67)	2.18 (1.78–2.58)	0.74
Feeling of wellbeing^9^	4.92 (4.55–5.30)	4.50 (4.15–4.86)	0.11

Missing: EQ-5D-3L domains that sum up in the Index: Mobility N = 15, Self-care N = 13, Usual activities N = 14, Pain/discomfort N = 14, Anxiety/depression N = 9, EQ-5D VAS N = 41.

Missing ESAS:

^1^N = 31,

^2^N = 31,

^3^N = 29,

^4^N = 23,

^5^N = 22,

^6^N = 23,

^7^N = 22,

^8^N = 21,

^9^N = 26.

## Discussion

In this prospective cohort study about the implementation of a structured care guide to facilitate integration of palliative care into geriatrics we found a significant improvement in patient satisfaction, particularly in the domains of decision-making, participation in care, communication, and information, while no difference was found in quality of life or symptom burden. This effect was seen after a short hospital stay and lasted several weeks after discharge in this vulnerable group of patients.

The overall results show that this complex intervention has been successful and has overcame some of the challenges reported in previous studies showing several barriers when integrating palliative care and geriatrics; limited knowledge and understanding of what the other discipline offers, difficulties where older adults fit in the health care system and lack of communication between disciplines [21,38,39]. The increased patient satisfaction in our study is in line with what should be expected when integrating palliative care into standard care. The aim of S-PCG is to improve patient-centred care through integration of a palliative care approach and patient-centred care has been associated with decreases in health care utilisation, increased patient satisfaction [40,41] and physical well-being, also among older people [41].

An important aspect of our results is the experience of increase in shared decision-making. Among older adults, shared decision-making is more complex due to possible cognitive impairment and tendency among some older adults to let family members or physicians make the decisions [42]. Thus, our results are encouraging, showing that involvement in decision-making is facilitated by the use of the S-PCG part 2 in geriatric care, even though further studies are needed to specifically assess the effect of S-PCG among patient with cognitive impairment. In our study the implementation S-PCG part 2 increased the experience of good communication and information, which helps to build trust, enables symptom control, strengthens coping and improves decision-making [43]. As similar results have been shown when palliative care was integrated in other specialities, this study indicates that the expected improvement in communication between patients and HCPs are also applicable for geriatric patients.

In the present study there was no significant improvement in HRQOL and symptom burden. In general, increase in HRQOL and improved symptom control have been shown when patient-centred care has been used [40]. However, hospitalized older adults exhibit a broad spectrum of symptoms, and they are likely to retain symptoms that were present at admission also after discharge [44]. The short in-hospital stay and the relatively long time period from discharge to filling in the questionnaires may have resulted in a return of previous symptoms especially since many of these patients lack structured and timely follow-up from their general practitioner [44].

The results of the study do not indicate any negative effects of the implementation and use of the S-PCG part 2. This is an important finding as any tool that conceivably takes time from the direct clinical care could affect the care quality. Indeed, a previous study on implementation of a different end-of-life care plan in geriatrics showed mixed results; decreased satisfaction with care assessed by family carers but improved comfort assessed by nurses [20].

### Strengths and limitations of the study

Strengths of the study include the large number of patients and that the pre-intervention and interventions groups showed no significant differences regarding sociodemographic and clinical characteristics and thus are comparable. Further, we reached a high response rate, showing that despite belonging to a frail group these patients want and are able to participate in a study based on questionnaires after discharge. In addition, as the study was performed at one hospital, the researchers had the possibility to be present at the wards weekly and thus all patients, without exceptions, were screened for participation.

There are also several limitations. The COVID-19 pandemic started during the intervention period and the patients during this period were therefore contacted by phone, which could potentially affect which patients who decided to participate. However, we saw no sign of a difference when comparing the two groups of patients. Also due to the pandemic, next of kin were not allowed at the ward during majority of the intervention part and they were not asked to participate. Thus, we cannot draw definite conclusions regarding next-of-kins experience. Further, generalisability might be affected as the study was performed at a single institution, even though the catchment area is the whole capital region, which includes more than 2 million people. In addition, patients with cognitive impairment were not possible to include due to the method with questionnaires after discharge. The S-PCG has been used in nursing homes among patients with cognitive impairment and is assessed by staff to be a valuable tool also in this group of patients. Further studies are ongoing to evaluate the effect of S-PCG among patients with cognitive impairment.

### Conclusions

The most important conclusion based on our results is that it was possible to introduce and implement a structured guidance for palliative care, such as the S-PCG part 2, into geriatric care. This resulted in improved care, especially in communication and shared decision-making, although it required a substantial effort in education, follow-up, and coaching. With the present model of implementing S-PCG part 2 some of the barriers can be overcome and make palliative care an integrated part of usual care and thus available to the increasing population of older adults with multimorbidity and frailty.

## Supporting information

S1 Table Next-of-kin satisfaction with care, measured with ten questions from the Swedish National patient survey. Results were compared between pre-intervention and intervention period.
(DOCX)

S1 Data (XLSX)
