# Supplementary material for: Increased patient satisfaction by integration of palliative care into geriatrics—A prospective cohort study
Source: PLoS One. 2023 Jun 22;18(6):e0287550. doi: 10.1371/journal.pone.0287550 (PMC10286968; doi:10.1371/journal.pone.0287550)
Supplement: S1 Table — Results were compared between pre-intervention and intervention period. (DOCX) [file pone.0287550.s001.docx]

**S1 Table**. Next-of-kin satisfaction with care, measured with ten questions from the Swedish National patient survey. Results were compared between pre-intervention and intervention period.

| **Next of kin** | **Pre-intervention**  (n=86)  Mean, (CI) | **Intervention**  (n=42)  Mean, (CI) | p-value |
| --- | --- | --- | --- |
| Did the HCPs consider your relative´s experience of his/her state of health?^1^ | 3.95  (3.74-4.16) | 4.07  (3.80-4.34) | 0.49 |
| If you, during your relatives stay, spoke to several HCPs, were they consistent regarding their communication?^2^ | 3.86  (3.63-4.09) | 4.15  (3.82-4.48) | 0.14 |
| Did you have the possibility to ask the questions you wanted?^3^ | 3.69  (3.39-3.99) | 3.90  (3.49-4.32) | 0.41 |
| If you asked HCPs questions, did you receive answers you understood?^4^ | 3.95  (3.77-4.13) | 4.20  (3.95-4.44) | 0.13 |
| Did HCPs involve you in decisions regarding your relative´s care/treatment?^5^ | 2.85  (2.52-3.16) | 3.36  (2.92-3.79) | 0.07 |
| Did you receive enough information about your relative´s care/treatment?^6^ | 3.08  (2.77-3.40) | 3.55  (3.15-3.95) | 0.08 |
| Did you receive enough information where to turn if you needed help or had additional questions after your relative´s stay?^7^ | 3.20  (2.87-3.54) | 3.95  (3.55-4.35) | **<0.01** |
| Do you think that the HCPs on the ward coordinated your relative´s contacts with the health care in the extent he/she needed?^8^ | 3.71  (3.41-4.01) | 4.07  (3.70-4.44) | 0.15 |
| Did the HCPs take into consideration your relative´s home/family situation when planning his/her discharge?^9^ | 3.77  (3.47-4.08) | 3.98  (3.52-4.43) | 0.46 |
| Did you feel safe during your relative´s stay in the ward?^10^ | 4.60  (4.45-4.76) | 4.83  (4.68-4.99) | 0.06 |

CI: confidence interval

HCP: Health-care professional

Missing: ^1^N=4, ^2^N=11, ^3^N=6, ^4^N=2, ^5^N=1, ^6^N=2, ^7^N=2, ^8^N=4, ^9^N=3, ^10^N=3.
